# Does Knee Arthroplasty Have a Beneficial Effect on Return to Work in Patients with Knee Osteoarthritis who Receive Long-Term Disability Benefits in the Netherlands?

**DOI:** 10.1007/s10926-024-10234-7

**Published:** 2024-09-10

**Authors:** Titi J. Cheng, Karen Nieuwenhuijsen, P. Paul F. M. Kuijer

**Affiliations:** 1 Amsterdam UMC Location University , of Amsterdam, Public and Occupational Health, Meibergdreef 9, Amsterdam, The Netherlands; 2 Research Center for Insurance Medicine (KCVG), Amsterdam, The Netherlands; 3 Dutch Employee Insurance Agency (UWV), La Guardiaweg 94-114, Amsterdam, The Netherlands; 4 Amsterdam Public Health, Societal Participation & Health, Amsterdam, The Netherlands; 5 Amsterdam Public Health, Quality of Care, Amsterdam, The Netherlands; 6 Amsterdam Movement Sciences, Musculoskeletal Health, Sports, Amsterdam, The Netherlands

**Keywords:** Osteoarthritis, Knee, Arthroplasty, Replacement, Knee, Return to work, Sick leave, Prognosis, Insurance, Disability

## Abstract

**Purpose:**

Optimal timing of knee arthroplasty (KA) is complex: operating at a younger age increases life time risk of revision, while delay results in an increased risk of job loss. This study evaluates whether disability benefits recipients due to knee osteoarthritis have an increased odds of returning to work (RTW) following KA.

**Methods:**

A retrospective cohort study was performed among long-term disability benefits recipients due to knee osteoarthritis using data of the Dutch Employee Insurance Agency. Logistic regression assessed whether recipients with KA had a higher odds of RTW in 10 years following start of disability benefits, compared to those without KA.

**Results:**

A total of 159 participants were included. During 10-year follow up, 42% had received KA and 37% had returned to work. No association was observed between KA and RTW (OR 1.39, 95% CI 0.62–3.12). Prognostic factors for RTW were being the main breadwinner (OR 7.93, 95% CI 2.95–21.32) and classification as 100% work disability (OR 0.20, 95% CI 0.09–0.45).

**Conclusions:**

KA has no beneficial effect on RTW among patients with knee osteoarthritis granted long-term disability in the Netherlands. For RTW, KA is probably best performed within the two years of paid sick leave before long-term disability is assessed in the Netherlands.

## Introduction

Knee osteoarthritis (OA) is one of the most common large joint disorders; the prevalence of symptomatic knee OA being estimated at 4.3% of the Dutch population in 2021 [1], and around 3.8% of global population in 2010 [2]. As annual incidence increases with age, peaking in the age group between 55 and 64 years [3], so does the prevalence in the older age groups. The disease results in decreased mobility and functioning and is associated with an increased risk of sick leave and loss of work [4]. Those working jobs that involve heavy lifting, kneeling, climbing stairs or ladders or kneeling and squatting combined with heavy lifting are at an increased risk of developing knee OA [5]. Which in turn leads to an increased risk of work disability, since these knee intensive activities are embedded in their working days [6]. Knee arthroplasty (KA) is proven effective in reducing pain and increasing mobility [7].

The number of surgeries has increased sharply over the past 20 years: the incidence rate per 100,000 population has more than doubled in the US and many other OECD countries, and especially among patients of working age [8, 9]. Even though surgical treatment of knee OA is generally effective and safe, one of the more complex issues that remains is optimal timing of surgery. Possibilities for revisions are not limitless. Younger patients have shown to have a significantly increased lifetime risk of revision [10, 11]. For many younger patients initial non-operative treatment will be recommended, justifiably so.

Despite good arguments to recommend non-operative treatment, delay of treatment comes with a prize: it can for instance lead to job loss. In turn, the loss of a job introduces risk factors that are known to be detrimental to health: loss of income, a less structured daily life, diminished social network and lower daily activity levels [12, 13]. These factors all lower the social economic status and thus damage the longer-term health: even healthy 50-year-old with lower incomes will have a noticeably worse health condition by the time they reach the age of 70, compared to those with higher incomes at baseline age [14].

The effects of KA on RTW and relevant prognostic patient factors have been frequently investigated. The percentage of RTW after KA varies between 68 and 85% [15–22]. Average time of RTW ranges from eight to twelve weeks after KA [22]. In short, those who receive KA quickly after being sick-listed have a favorable chance of returning to their original job. It is as yet unknown whether a similar positive effect can be found in patients with knee OA that are receiving long-term disability benefits. Under many circumstances these former workers lack actual work places to return to, thereby facing a bigger barrier to returning to the working population.

The aim of this study is therefore to determine whether having received KA correlates with increased chances of RTW among those receiving long-term disability benefits due to knee OA.

## Methods

The reporting in this paper follows the recommendations of the STROBE statement.

### Work Disability Context in the Netherlands

This study was performed in the Netherlands. During the first two years of sick leave, both employee and employer are responsible for achieving RTW [23]. The employer is assisted by the occupational physician to do so. After two years of being sick-listed, while being paid by the employer, the Dutch Employee Insurance Agency (EIA) assesses whether the employee meets the criteria for receiving disability benefits [24].

Eligibility for disability benefits in the Netherlands is determined by loss of income capacity, expressed as a percentage of the insured income. Through a convoluted system matching the remaining functional capacity with possible work options, a theoretical remaining income capacity is determined. The theoretical loss of income capacity is therefore a function of physical and mental functional limitations caused by disease, and also relates to educational level and insured income. Employees who have higher attained educational levels might be considered able to work jobs that merely require lower education. Yet the inverse situation does not apply: a lower educated employee cannot be expected to earn their income by working a job that they do not have the proper qualification for. At the same time, higher educated workers tend to have higher insured incomes. This higher reference point might result in a bigger proportional loss of income if disability forcibly results in a career change, often into entry-level jobs. To be eligible for disability benefits the loss of income needs to be at least 35%. Any loss of income over 80% is considered equal to 100% and is consequently classified as ‘100% work disability’. In some cases this 100% work disability might be considered permanent, for instance due to progressive or untreatable chronic diseases. In those cases, no further work rehabilitation will be supported or attempted, nor will future disability evaluations will be performed. This permanent disability retirement is called IVA, and comes with an additional 5% increase in benefits pay out (up to the regular ceiling of 75% wage replacement).

Once the sick-listed employee has been granted disability benefits, the employer can apply for permission to terminate the original employment contract, leaving the recipient of disability benefits with no actual work place to return to. For some employees, employment continues under different conditions, such as other primary job responsibilities, adjusted working circumstances, or reduced number of working hours. However, the employer is not obligated to provide such an arrangement. When a worker is no longer employed by the former employer, RTW is usually achieved through applying for a new job and being hired by a new employer. In most cases this also constitutes a career change, since the recipient of disability benefits typically remains unable to perform their original job.

### Study Design and Participants

A retrospective cohort study was performed among participants who receive disability benefits in the Netherlands. With prior permission and cooperation from the Dutch Employee Insurance Agency, recipients of disability benefits received a letter asking them to voluntarily participate in this study with no effect on their benefits whatsoever. The letters were sent out to participants who met the following inclusion criteria: having been granted disability benefits between 2005 and 2010; primary cause for sick leave being knee OA; age at start of disability benefits being between 18 and 57 years. The cut-off limit for inclusion of 57 years was chosen in order to allow for completion of a 10-year follow-up period after the start of disability benefits. The retirement age in the Netherlands was approximately 66.33 years in 2020 [25].

Exclusion criteria were: *permanent* 100% disability status (IVA); registered diagnosis codes corresponding to severe comorbidities, namely malignancy, heart failure, cerebrovascular accident, multiple sclerosis, schizophrenia, and rheumatoid arthritis. The mentioned severe comorbidities are known to result in 100% disability status (either permanent or non-permanent) in more than half of the cases where one of these diseases is present [26]. As such, in those cases any resulting disability should be mainly attributed to those diagnoses and probably not to knee OA.

T_0_ has been defined as the moment when disability benefits commenced. T_10_ is defined as 10 years later. The cut-off limit for the age of 57 years also approximately coincides with the age range of 55 to 60 years which is used in several studies to consider the lifetime risk of revision for KA [11, 27, 28].

The participants filled out a questionnaire after having provided informed consent. The procedure and explanation of explicitly voluntary participation was explained in the accompanying letter. As said, either accepting or declining to participate would have no effect whatsoever on their disability benefits status. Participants who had not fully completed the questionnaire were sent a second reminding letter requesting completion of the questionnaire. Relevant covariates that might influence the primary outcome RTW in relation to KA were identified in two systematic reviews and a cohort study [16, 19, 29]. Questions regarding these covariates were included in the questionnaires.

The results from the questionnaires were linked to an anonymized database from the Dutch Employee Insurance Agency encompassing age at T_0_, year at which T_0_ occurred for each individual, sex, status of partial (< 80%) or 80–100% disability.

Both sets of anonymized data were connected using an intermediate participant ID, ensuring that the investigators were unable to trace the submitted answers back to the individual participant.

The analyzed follow-up period is between start of disability benefits and the following ten years. Any type of RTW occurring outside of this period was disregarded.

### Questionnaire

The questionnaire consists of items describing RTW, the independent variable KA and the predefined covariates.

#### Return to Work

RTW was defined in the following manner: whether participants had performed any paid work between T_0_ and T_10,_ whether this lasted a minimum of 26 consecutive weeks and whether they had worked on average a minimum of five working hours per week. Participants were also asked which year any RTW occurred. Any paid work was taken into account, including work other than the original occupation before start of disability benefits. Work as a contractor or self-employed also qualified as RTW, as long as all of the above mentioned criteria were fulfilled. These criteria coincide with the current Dutch criteria in order to receive unemployment benefits [30].

#### Knee Arthroplasty

The study participants were asked whether they had received knee replacement surgery after T_0_, and in which year(s) they had received KA, Participants were not asked to make the technical distinction between unicompartmental knee arthroplasty (UKA) and total knee arthroplasty (TKA). Therefore, the study is unable to distinguish whether participants had received UKA or TKA. Similarly, no distinction was made between primary or revision procedures. For brevity, all types of knee procedures will be referred to as KA.

#### Covariates

The questionnaire contained the following questions for the participants: validating whether knee OA was the primary cause resulting in disability benefits (binary, yes or no), their highest attained education level (multiple choice, ordinal), their last occupation before sick leave occurred (open question, nominal), whether the participants thought that this job was physically demanding (multiple choice, nominal), whether participants were the main breadwinner of their household at the time of being sick-listed (binary), current body height and weight (numeric), and relevant weight changes between T_0_ and T_10_, defined as 10% or more weight change in the ten-year interval (binary). The current weight was combined with the reported weight change to estimate weight at T_0_.

The Work, Osteoarthritis or joint-Replacement Questionnaire (WORQ) was also included to assess the self-reported physical difficulty performing knee-demanding work-related activities. The WORQ is a validated questionnaire designed to evaluate the impact of knee complaints on participants’ ability to work following KA [31]. It consists of 13 questions regarding work-related activities that involve using the knee. Participants were asked to grade difficulty in performing these particular activities on a five-point scale. The total score is converted to a score between 0 and 100 (%), a score of 0 indicating perceived complete inability and a score of 100 corresponding to perceiving no functional inhibition by the knee. A score of 50% or lower can also be interpreted as dissatisfaction in knee performance, whereas a score of 71% or higher indicates general satisfaction in knee performance.

#### Defining Heavy Physical Work

When asked to categorize the physical demands of their former work, participants were given the choice between ‘light’, ‘moderately heavy’ or ‘heavy’ work. The list of job descriptions was also presented to two work specialists, in order to verify this categorization and to establish consistency across all the participants. If there was a disagreement between participants and work specialist, the classification from the latter prevailed.

### Data Analysis

**Fig. 1:**
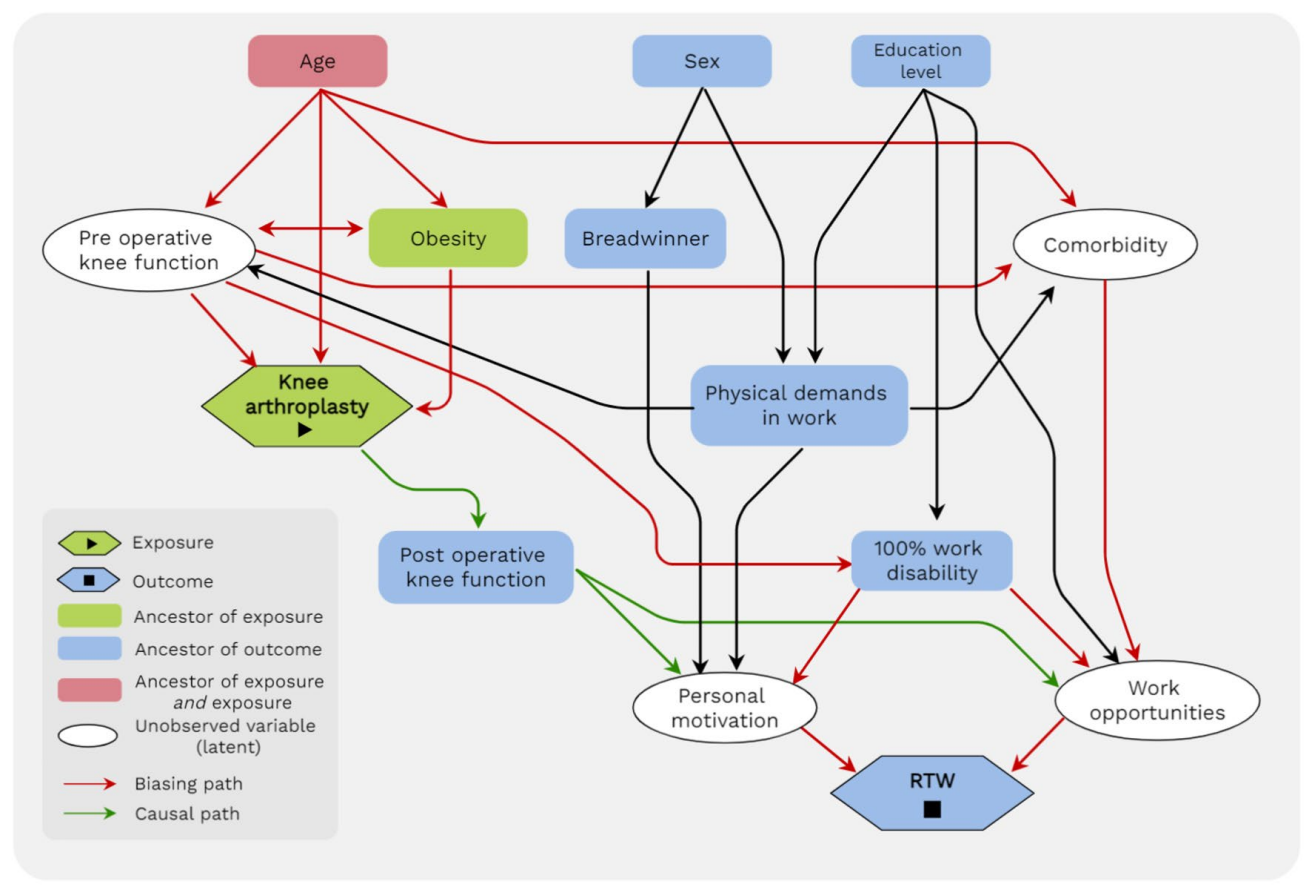
Directed Acyclic Graph showing causal relations between covariates deemed relevant for Return To Work among Dutch recipients of a disability benefit due to knee osteoarthritis

A Directed Acyclic Graph (DAG) was created depicting the relationship between outcome, exposure and relevant covariates. The covariates were identified from previous studies (DAG) [29]. This DAG was visualized using the DAGgity web tool v3.0 [32]. Through the DAG, confounders were identified that require minimal sufficient adjustment in order to make a reasonable estimate of the effect of KA on RTW [33]. Using the DAG approach an adequate adjustment for confounders is achieved without introducing over adjustment bias [34]. From the DAG (Fig. 1), the following variables were selected: age at T_0_, disability classification, previously heavy physical work and being the main breadwinner of the household.

Further statistical analysis was performed in IBM SPSS v26. Descriptive statistics were generated for analyzation of demographic data of the participants, covariates and RTW. A check on collinearity was performed using a cut-off limit of VIF > 2.5 indicating no significant collinearity was detected. Stepwise multivariable logistic regression was performed with RTW at the 10-year mark as the dependent variable. Independent variables are having received KA and the variables from the DAG. Odds ratios (OR) including a 95% confidence interval (CI) were calculated. A *p* value of < 0.05 was considered statistically significant.

## Results

### Participants

**Fig. 2:**
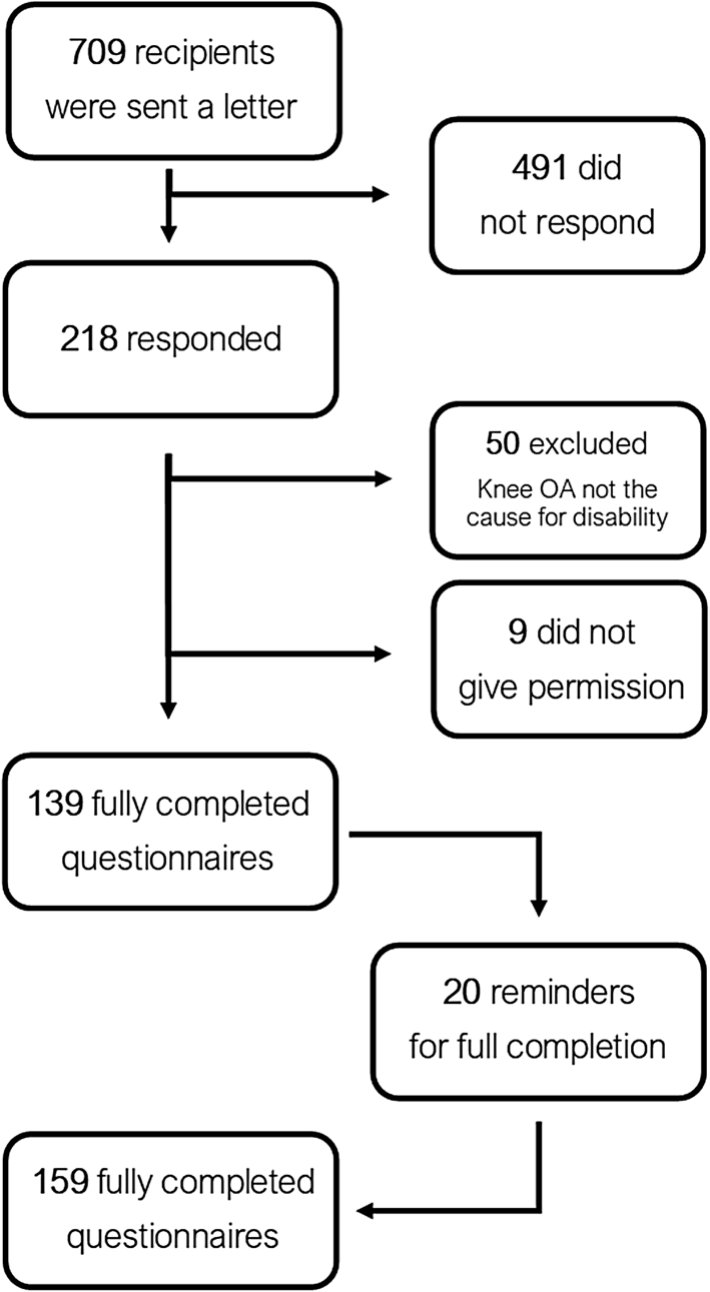
Flow chart of the inclusion of participants

A total of 709 potential participants receiving disability benefits due to knee OA were invited to participate. Of these, 218 people (30%) responded. Fifty respondents indicated that knee OA was in fact *not* the primary cause for sick leave and resulting disability benefits. Therefore, these participants were excluded. Nine participants responded and did not wish to participate in the study. Twenty participants returned questionnaires that turned out to be incomplete. After sending a reminding letter, all twenty were completed. For the final analysis, a total of 159 participants were included (Fig. 2).

### Descriptive Data

**Fig. 3:**
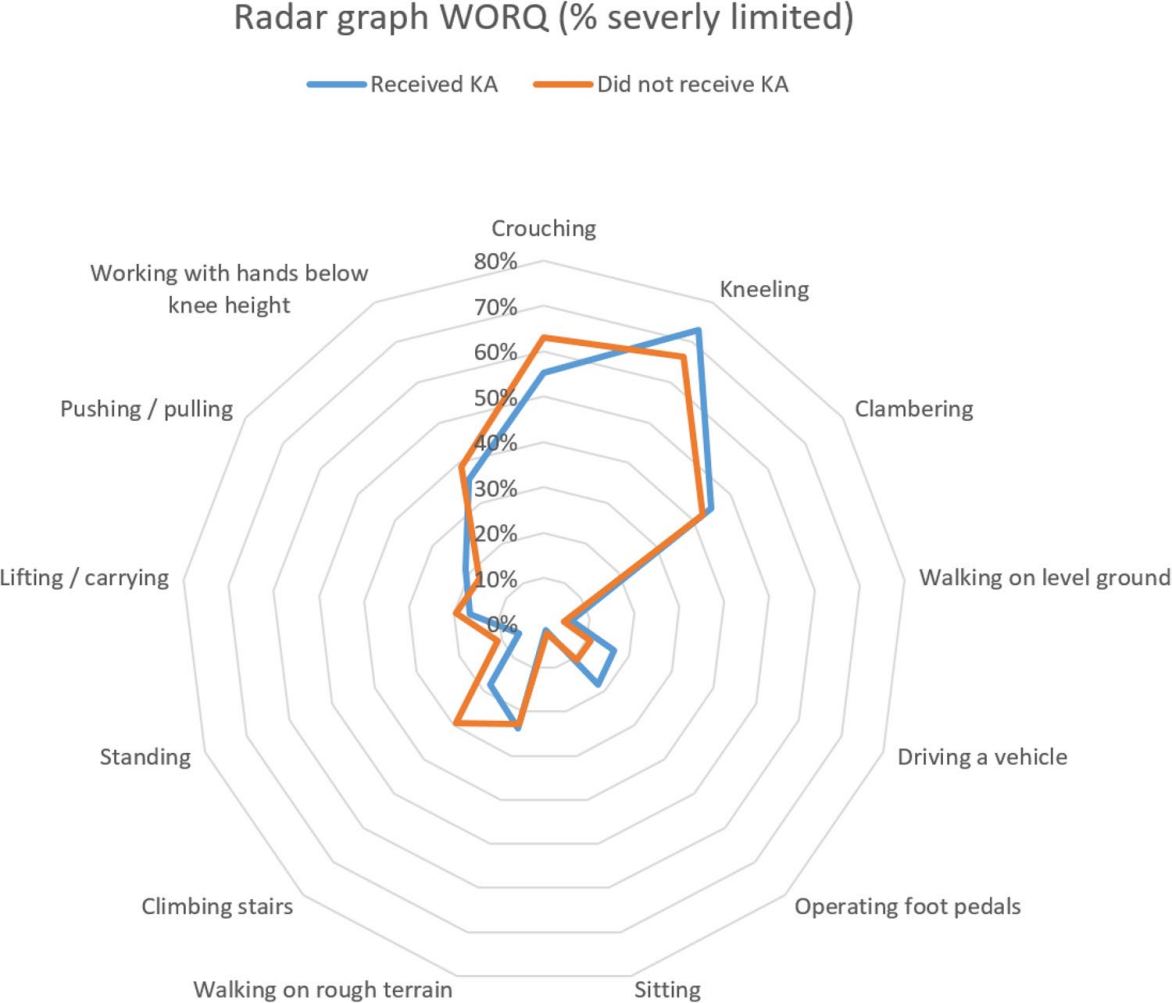
Radar graph showing percentage of participants reporting severe limitations in performing specific work-related activities

Out of 159 participants, 55% was male and 45% was female (Table 1). Median age was 51.0 years. Estimated median BMI at T_0_ was 28.4, 78% was estimated to be overweight (BMI ≥ 25 kg/m^2^). 35% of participants previously worked in heavy work, 53% in moderately heavy work and 13% in light work. 63% was the main breadwinner of the household at T_0_. 63% of all participants was evaluated at 100% work disability at T_0_. The current WORQ scores were determined: A total of 104 participants (65%) indicated a score of less than 50%, i.e. dissatisfaction with their knee functioning. Forty-five participants (28%) scored between 50–70%. Only ten participants (6%) scored above 70% and were satisfied with their knee functioning. In the 10-year interval between T_0_ and T_10_ 67 participants (42%) had received KA (Fig. 3).

**Table 1:** Characteristics of study participants (*n* = 159)

Sociodemographic variables	Entire study population (%) *n* = 159	Received KA (%) *n* = 67 (42%)	Did not receive KA (%) *n* = 92
*Sex*			
Male	88 (55)	31 (46)	57 (62)
Female	71 (45)	36 (54)	35 (38)
*Age at T*_*0*_^*a*^			
Median	51.0	51.9	50.3
< = 40 y	13 (8)	3 (4)	10 (11)
41—45 y	18 (11)	6 (9)	12 (13)
46—50 y	48 (30)	21 (31)	27 (29)
51—56 y	80 (50)	37 (55)	43 (47)
*BMI at T*_*0*_^*a*^			
Median (kg/m^2^)	28.4	28.5	27.9
< 25 kg/m^2^	35 (22)	14 (21)	21 (23)
= > 25 kg/m^2^	124 (78)	53 (79)	71 (77)
*Educational level*			
Elementary	19 (12)	9 (13)	10 (11)
Lower vocational	102 (64)	48 (72)	54 (59)
Intermediate vocational / higher general	34 (21)	9 (13)	25 (27)
Higher vocational / university	4 (3)	1 (1)	3 (3)
*Level of physical demands in previous work*			
Light	20 (13)	7 (10)	13 (14)
Intermediate	84 (53)	40 (60)	44 (48)
Heavy	55 (35)	20 (30)	35 (38)
*Main breadwinner*			
Yes	100 (63)	38 (57)	62 (67)
No	59 (37)	29 (43)	30 (33)
*Return to work*			
Yes	59 (37)	23 (34)	36 (39)
No	100 (63)	44 (66)	56 (61)
WORQ scores (%) at T_10_			
< 50	104 (65)	45 (67)	59 (64)
50 – 70	45 (28)	17 (25)	28 (30)
> 70	10 (6)	5 (7)	5 (5)

^a^T_0_ is defined as the start of disability benefits. T_10_ is 10 years later

### Prognostic Factors Associated with Return to Work

Out of 159 participants, roughly a third (37.1%, n = 59) indicated they had returned to paid work between T_0_ and T_10_. The regression analysis showed no significant difference between those that reported having received KA and those that did not (p = 0.42; 95% CI OR 0.62—3.12). Further analysis showed that being the main breadwinner of the household at T_0_ was positively correlated to future RTW: OR 7.93 (95% CI: 2.95 – 21.3). Inversely, those that were assigned 100% disability status at T_0_ were less likely to RTW (OR 0.20; 95% CI: 0.09 – 0.45). No relationship with RTW was observed for having a history of physically demanding work (*p* = 0.40; 95% CI: 0.43 – 1.4) or age at T_0_ (*p* = 0.12; 95% CI: 0.873 – 1.016), see Table 2.

**Table 2:** Logistic regression analysis of impact of predetermined covariates on RTW in disability benefits recipients due to knee osteoarthritis

	Odds Ratio	95% CI	*p*
Age at T0	0.942	(0.873 – 1.016)	0.123
100% work disability at T0	0.198*	**(0.087 – 0.451)**	**0.000**
Previous heavy physical work	0.776	(0.432 – 1.392)	0.395
Household breadwinner at T0	7.928*	**(2.949 – 21.315)**	**0.000**
Received TKA after T0	1.394	(0.623 – 3.122)	0.274

^*^statistically significant with *p* < 0.05

## Discussion

In our cohort of 159 participants, we compared RTW rates of those that received KA after the start of their Dutch disability benefits to those that abstained from KA. The overall chance of RTW in this cohort was 37%. No significant association was observed between having received KA and higher odds of RTW. However, other factors were related to RTW. Being the main breadwinner of the household during start of disability benefits turned out to be a positive prognostic factor for RTW. Inversely, being assigned the status of 100% disability during initial disability evaluation was negatively correlated to RTW.

These results align with findings from previous studies showing both the positive and negative impact of these factors [29, 35].

In this study, the overall RTW rate of approximately 4 in 10 seems low when compared to previous studies investigating the effect of KA on RTW, reporting rates of about 7 in 10 [21, 22]. However, these other studies were predominantly performed on workers shortly after being sick-listed, whereas the participants in this study had been sick-listed over two to ten years ago. This difference in RTW rates could suggest that the initial reasons for work disability might play a less pronounced role as time progresses and other factors gain relevance. Even though knee OA was the prime reason for initially being sick-listed in these participants, after more than two years of sick leave KA no longer results in an improved RTW. This is despite the known positive effect that KA might have on knee functioning and resulting work ability [16, 36, 37]. Therefore, the cascading effect of improved knee functioning into factual return to the working population seems fairly limited in workers who are already on long-term disability benefits.

These findings could be partially explained by the fact that a large proportion of our study cohort (a) had a low to very low educational level (76% either lower vocational or elementary), (b) previously performed work with intermediate to heavy physical demands (87%) and (c) was of relatively old age (median 51 years). These factors combined in a single person could pose a significant barrier into finding viable alternative work options [38, 39].

In the specific Dutch situation, being classified for 100% disability during initial evaluation could suggest an accurate disability evaluation. However, one should bear in mind that a certain degree of self-fulfilling prophecy comes into play. The disability benefits recipient could interpret the outcome of ‘100% disability’ as a confirmation that it is indeed pointless to even consider searching for work in the future. Previous studies have shown that patient expectations are an important prognostic factor for return to work after KA. And then there is also the difference in adjustments in the Dutch disability benefits regime. Workers who have a total disability classification suffer no financial penalties for not returning to work after a certain amount of time (months to years), in contrary to those that have a partial disability classification. For the latter, there is a fairly strong financial incentive to actively look for (alternative) work, in order to continue receiving the maximum of their benefits pay outs. Therefore, optimal timing of KA is not only important from a biological perspective but also from a psychosocial perspective to enhance quality of life [40], for which having paid work is of utmost importance [41]. Moreover, patients at risk for return to work after KA should be provided with work-directed care like for instance Goal Attainment Scaling [42] and other types of promising integrated care [43–46].

### Strengths and Limitations

The main strength of this study is the long follow-up period of 10 years. Participants were selected through the Dutch disability system, having received a reliable evaluation of their work disability by a trained medical specialist. Standardized diagnose codes are used within this system, enabling a broad selection of participants whose main reasons for receiving disability benefits was having knee OA. On the other hand, it is unknown what motivated the study participants to respond to the invitation and why others did not.

Even though the study design was retrospective, we believe that both receiving KA and returning to the working population are such major events that the answers in the questionnaire are accurate and hardly open for interpretation, thereby limiting the risk of recall bias. In fact, most of the other collected covariates from the questionnaires were reliably assessed and left little room for personal opinion or interpretation. The WORQ is a good indicator of patient satisfaction regarding their knee functioning in work activities. RTW was well defined and excluded incidental short working periods (e.g. lasting only several days) through the use of multiple questions on the questionnaire.

Due to the specific setting of this study in the Netherlands with the Dutch disability benefits system, generalizing should be done cautiously. The Dutch disability benefits system is designed to incentivize finding alternative work options, introducing a certain survivorship bias. In general, lower educated workers have fewer alternative career options that rely less on physical workload. Among these people, more severe physical limitations therefore lead to increased odds of qualifying for disability benefits. Inversely, it is more likely that lower educated workers with fewer physical limitations and higher chances of finding alternative work, have been filtered out by the disability benefits evaluation process already and were therefore not included in this study.

The same principle applies for higher educated workers that might have more severe knee problems but remain able to earn a similar income (compared to before sick-listed), simply due to the fact that they are more likely to find alternative work that doesn’t require a well-functioning knee.

Furthermore, certain elements that undoubtedly impact RTW have been ignored for simplicity’s sake. For instance psychiatric comorbidity, other significant physical comorbidity introduced *after* the start of disability benefits (e.g. occurrence of stroke of myocardial infarction), personal motivation, job market differences between major industry sectors, or regional differences. Taking into account for most or all of these examples would have made statistical analysis nearly impossible due to demands for increased sampling size.

### Implications and Recommendations

This study was not able to determine an effect of KA on RTW among those receiving long-term disability benefits due to knee OA. This provides a stark contrast to previous studies that focused on the relatively short term, and which showed decent to good rates of RTW among those that receive KA *early* after getting sick-listed [36]. The current findings do not contradict earlier findings, but instead only confirm the hypothesis that an early intervention to perform KA probably yields the best results in regards to RTW. Within the Dutch disability benefits system, it seems much easier to retain one’s job after early KA than it is finding a new (or other) job after job loss due to severe knee OA once two years of sick leave have passed.

Therefore, treating physicians should also take into account the long-term negative effects on health status that comes along with prolonged disability, lack of work participation, decreased social activities and decreased physical exercise. Of course, we do realize that there are many more factors other than RTW to consider before deciding whether the time is right for KA, but hopefully this study illustrates the limited window of opportunity for preserving patients for the working population.

Future studies into effective occupational rehabilitation after KA might find improved importance and urgency, knowing that cost efficiency of early KA is increased when the long-term costs of disability benefits and compounded negative health outcomes are also taken into consideration [47].

## Conclusion

This retrospective cohort study showed that only four out of ten recipients of long-term disability benefits due to knee osteoarthritis managed to return to paid work in the ten years following initial disability evaluation. Having received a knee arthroplasty did not result in an improved return to paid work. Being a breadwinner improved the likelihood of having paid work and receiving 100% work disability benefit reduced this likelihood. Therefore, more attention should be given to optimal timing of KA, including enhanced postoperative occupational rehabilitation.

## Data Availability

The datasets generated during the current study are available from the corresponding author upon reasonable request.
